# Length of the Adult Human Colon in Health and Constipation Measured Using Magnetic Resonance Imaging

**DOI:** 10.1111/nmo.70215

**Published:** 2025-12-02

**Authors:** Faiz Alqarni, Soma Kumasaka, Caroline L. Hoad, Victoria Wilkinson‐Smith, Stuart Taylor, David Atkinson, Iyad Naim, Alex Menys, S. Mark Scott, Marc A. Benninga, Hayfa Sharif, Penny A. Gowland, Moira A. Taylor, Guruprasad P. Aithal, Robin C. Spiller, Maura Corsetti, Luca Marciani

**Affiliations:** ^1^ Translational Medical Sciences, Nottingham Digestive Diseases Centre, School of Medicine University of Nottingham Nottingham UK; ^2^ National Institute for Health and Care Research (NIHR) Nottingham Biomedical Research Centre Nottingham University Hospitals NHS Trust, and University of Nottingham Nottingham UK; ^3^ King Saud Medical City, Ministry of Health Riyadh Saudi Arabia; ^4^ Department of Diagnostic Radiology and Nuclear Medicine Gunma University Graduate School of Medicine Gunma Japan; ^5^ Radiological Sciences, School of Medicine University of Nottingham Nottingham UK; ^6^ Sir Peter Mansfield Imaging Centre, School of Physics and Astronomy University of Nottingham Nottingham UK; ^7^ Centre for Medical Imaging University College London London UK; ^8^ Motilent Ltd London UK; ^9^ Blizard Institute, Queen Mary University of London London UK; ^10^ Emma Children's Hospital Department of Pediatric Gastroenterology, Amsterdam UMC Amsterdam the Netherlands; ^11^ Jaber Al‐Ahmad Armed Forces Hospital, Ministry of Defence Kuwait City Kuwait; ^12^ School of Life Sciences University of Nottingham Nottingham UK

**Keywords:** chronic constipation, colon length, gastrointestinal imaging, macrogol, MRI

## Abstract

**Background:**

Quantitative data on colon length in adult chronic constipation (CC) are lacking. This study aimed to measure the length of the colon in CC, in the undisturbed state and after an osmotic laxative challenge, using magnetic resonance imaging (MRI) as compared to healthy volunteers (HV) and IBS‐C patients.

**Methods:**

Segmental and total colon length were measured by manual tracing on fasting MRI scans, retrieved retrospectively for 57 HV, 17 CC, and nine patients with irritable bowel syndrome with constipation (IBS‐C). In all CC patients and 22 HV, MRI scans were also performed after an oral osmotic laxative challenge. Participants' age range was 18–75 years.

**Key Results:**

CC patients showed significantly longer colons (162 ± 6 cm) than HV (127 ± 2 cm; *p* < 0.01), with 10/17 being longer than the upper limit of normal. Colon length in IBS‐C (129 ± 6 cm) was similar to HV. The colon in HV was able to elongate from 133 ± 3 to 148 ± 4 cm (*p* < 0.0001) to accommodate the macrogol challenge influx, while the CC colon could not do so (from total length at baseline 162 ± 6 to 168 ± 5 cm; *p* = 0.0768).

**Conclusion & Inferences:**

The study provides normative values of colon length, to which CC and IBS‐C are compared. CC was associated with increased colon length and reduced capacity to elongate longitudinally, rather than radially, in response to a laxative challenge. Colon length in IBS‐C was similar to HV. These measurements can improve our understanding of gut disease pathophysiology and response to treatment.

## Summary

- Magnetic resonance imaging (MRI) can measure colon length in the physiological state, without the use of any contrast agent via oral or rectal route.
- Colon length is significantly longer in adult chronic constipation than in healthy volunteers and in irritable bowel syndrome with constipation.
- The chronically constipated colon is unable to accommodate longitudinally in response to fluid influx from a macrogol challenge.

## Graphical abstract

Colon length was measured in adult healthy volunteers, people with chronic constipation, and people with IBS with constipation using MRI. Colon length is significantly longer in chronic constipation. The chronically constipated colon is unable to accommodate longitudinally in response to fluid influx from a macrogol challenge.

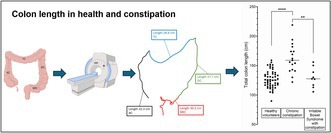

## 1 Introduction

The human colon plays a fundamental role in gastrointestinal physiology and is primarily responsible for water, electrolyte, short‐chain fatty acid, and some vitamin and mineral absorption, stool formation, and the regulation of defecation. Alterations in its structural integrity or functional capacity can contribute to the development of gastrointestinal disorders, with chronic constipation (CC) being one of the most common [1, 2, 3, 4, 5]. The literature describes observations from autopsies, endoscopy, and radiology of elongated colon in CC, with tortuous (dolichocolon) variants [6, 7, 8, 9, 10]. Quantitative knowledge of colon length in health and disease is, however, limited. Traditionally, colon length measurements [11], were obtained from cadaver studies [12], or in vivo using various diagnostic techniques that are either invasive such as intubation [13], require colon cleansing such as catheter‐tracking endoscopy [14], or prepare and distend the bowel with large amounts of luminal contrast media such as barium enema [15], and computed tomography, which can markedly alter the resting state morphology [9], and also expose patients to ionizing radiation. MRI can offer a noninvasive alternative, providing high‐resolution images of the undisturbed colon without bowel preparation or ionizing radiation exposure. A comparative study using MRI and an electromagnetic tracking system measured colon length in 25 healthy adults [16]. The MRI method involved segmenting the colon images and then applying a mathematical algorithm to determine and measure the centerline (medial path) through the segmented colon, a process called 3D topological skeletonization. This study provided initial normative MRI values for different anatomical segments of the colon, with a mean total colon length from ascending to rectosigmoid colon of 95 cm and a standard deviation of 15 cm. A subsequent study used similar MRI methods to study colon length in children with and without constipation [17]. The findings indicated that children with CC had significantly longer colons than HV, particularly in the ascending and sigmoid regions. The skeletonization method requires colon segmentation, which can be laborious; the skeletonization algorithm can also suffer from missing connections between colon segments due to image or segmentation imperfections and can branch out or “jump” between tortuous segments [17]. Data in adults with CC are also lacking.

Based on these considerations, this study aimed to measure the length of the colon and of its anatomical segments from MRI imaging using a manual centerline “node dropping” technique and test the hypotheses that: (1) the length of the colon is longer in adult patients with CC compared to HV and IBS‐C (2) the colon length response to an oral osmotic laxative treatment challenge is different between CC and HV. The study also aimed to assess the reproducibility of the MRI colon length measurements carried out using the manual centerline node dropping technique.

## 2 Methods

### 2.1 Participants

Retrospective MRI image datasets were retrieved for 57 adult HV with no symptoms of constipation from the RECLAIM [18], MAGIC [19], APPLE [20], KIWI [21], and MRLENGTH (unpublished) studies. For the first aim, equivalent retrospective colon MRI image datasets were also retrieved for 17 adult patients with CC (the Nottingham cohort of the RECLAIM study [18]). The patients fulfilled the Rome IV criteria for functional constipation [1]. MRI datasets were also retrieved for nine patients with IBS‐C. The latter were the IBS‐C adult cohort of the EFIGI [22] study, with the IBS subtype confirmed according to the Rome IV criteria [1].

For all participants, the baseline MRI datasets of the colon were acquired after an overnight fast, in a physiological, undisturbed state without the use of any oral or rectally administered luminal contrast agent. The datasets were acquired on a range of 1.5 T and 3 T scanners employing a breath‐hold, 3D, coronal, dual echo fast field echo sequence to provide a complete view of colonic anatomy and enable measurement of colon length. Typical MRI parameters for the sequence at 3 T: echo times out of phase/in phase = 1.15 ms/2.30 ms; repetition time = 110 ms; 60°flip angle; 22 coronal slices; acquired with voxel size 1.6 × 1.6 × 8 mm^3^.

To test the second hypothesis (that colon length response to an oral osmotic laxative treatment challenge is different between CC and HV), retrospective data from all the above‐mentioned 17 CC patients and 22 of the HV who underwent an osmotic laxative challenge after the fasting baseline scan as part of the RECLAIM [18], study protocol.

Finally, to assess the variability of the colon length measurements, a subset of IBS‐C patients who had a fasted colon baseline MRI scan four times on four separate occasions [22], was also retrieved.

The above‐mentioned studies all had Ethics approval, namely: RECLAIM study [18], approval number 17/EM/0032; MAGIC study [19], approval number 17/WM/0049; APPLE study [20], approval number A13102015; KIWI study [21], approval number A200317; MRLENGH study approval number FMHS 204–0223; EFIGI [22] study, approval number 17/EM/0277. All participants provided written informed consent.

### 2.2 MRI Image Analysis

**FIGURE 1:**
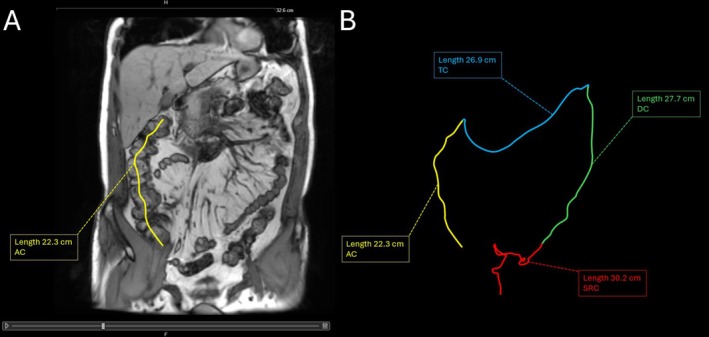
(A) Example of length measurements for the ascending colon (AC) segment of a participant. The figure displays one MRI coronal image plane of the multi‐slice stack with the yellow AC length measuring line projected on top. (B) The measuring lines for the entire colon length, comprising the four individual measuring lines for the AC (in yellow), transverse colon (TC, in blue), descending colon (DC, in green), and sigmoid‐rectum colon (SRC, in red). The measuring lines are manually drawn in three dimensions on the coronal multi‐slice MRI stack and displayed projected on the two‐dimensional plane.

MRI images were analyzed using the Entrolytics platform (Motilent, London, UK), a tool specifically designed for gastrointestinal imaging analysis. Using this platform, the operator uploaded the coronal image dataset for each participant. The images were first viewed to familiarize himself with the anatomy of the participant. The operator then manually defined the centerline of each colonic segment in 3D, including the ascending colon (AC), transverse colon (TC), descending colon (DC), and sigmoid rectum colon (SRC) (Figure 1). The process began with marking the beginning of the colon at the cecum and placing, with a click on the workstation mouse, the first node at the bottom of the cecum. After the placement of this first node, the operator scrolled through the coronal images, identifying visually the centerline of the colon segment and dropping sequentially centerline nodes to mark the AC's centerline in 3D, proximally, until the hepatic flexure was reached. This served as the anatomical landmark indicating the end of the ascending colon. The operator then started a second centerline from that point, dropping sequentially centerline nodes to mark the TC's centerline in 3D until the splenic flexure was reached. This process was then repeated for the DC, marking a new centerline distally until the pelvic brim, where the colon begins to change direction. At this point, the operator started the last centerline to mark the SRC from this point, extending distally through to the anal region. Total colon length is then calculated as the sum of the four segments for each participant. The centerline tool on the platform accounts for any variability in slice thickness, gap, and spacing between the scans and automatically generates a real‐world 3D measurement of each segment in cm, to a 0.1 cm resolution. The operator was not blind to the different groups.

### 2.3 Colon Length in the Fasting Condition

Colon length measurements were carried out for the four anatomical segments, and the total colon length was taken as the sum of the four individual segments for a participant.

### 2.4 Colon Length Response to an Osmotic Laxative Challenge

Each participant in that study, after fasting baseline (T0) MRI acquisition, received a dose of MoviPrep (Norgine), calculated as 10 mL per kilogram of body weight, with a total volume ranging between 500 and 1000 mL depending on individual weight. MoviPrep contains macrogol and electrolytes, which work by drawing water into the bowel lumen and stimulating motility. MRI scans acquired 120 min after ingestion (T120) of the macrogol challenge were analyzed to compare with T0 colon length. The time point at 120 min was chosen rather than the other time point available at 60 min, as from the original paper, it can be seen that the full effect of the challenge took place at T120 [18].

From the original RECLAIM study [18], MRI dataset, we were also able to retrieve the values of total colon volume for the CC patients and HV, which were measured by manually segmenting the datasets. The colon volume image analysis had been carried out according to published methods [23], whereby regional boundaries were identified by anatomical landmarks such as the superior points of the hepatic and splenic flexures and the point where the descending colon deviates posteriorly or medially. The colon regions were then traced manually within each coronal MRI image slice, building a 3D representation of the morphology, thus allowing segmental volume measurement of each region. The availability of colon volume data and comparison with the colon length measurements allowed inference on the colon longitudinal and axial accommodation to the osmotic laxative challenge.

### 2.5 Intraoperator and Within‐Subject Variability

Intraoperator variability for the new measurement technique was assessed on one IBS‐C participant, randomly selected. Colon length measurements were performed on six different occasions by the same operator, and the coefficient of variation (CoV) was calculated for each colonic segment and total colon length.

Within‐subject variability of colonic length with time was evaluated in eight consecutive IBS‐C patients from the EFIGI study [22]. These patients had a fasting MRI scan acquired once a week for four consecutive weeks, allowing the assessment of potential natural variation in colonic length. The CoV was again calculated for each colonic segment and for the total colon length.

### 2.6 Data Analysis and Statistics

GraphPad Prism (version 10.3.1, GraphPad Software, San Diego, CA, USA) was used to calculate basic descriptive statistics, analyze, and plot the data. Normality of the data was assessed using Shapiro–Wilk's test. Segmental and total colon lengths were compared between groups using an unpaired two‐tailed t‐test for normally distributed data and a Mann–Whitney test for non‐normally distributed data. Possible correlations between total colon length and demographic characteristics were explored using linear regression. The Upper Limit of Normal was defined as the mean plus 1.96 standard deviations. The coefficient of variation (CoV) was calculated as (mean/SD) × 100 to assess variability. A significance threshold of *p* < 0.05 was applied to determine statistical differences. Statistical inferences were corrected using the multiple comparisons Bonferroni correction unless explicitly indicated. The results are presented using the mean and the standard deviation (SD) or standard error of the mean (SEM) as indicated.

## 3 Results

Participants were predominantly female, with a broad age range from 18 to 75 years old. Summary demographic characteristics for each group are listed in Table 1.

**TABLE 1:** Participants' demographics and colon length measurements. Values are presented as mean ± SEM.

	Healthy volunteers	People with chronic constipation	People with irritable bowel syndrome with constipation
*n*	57	17	9
Age (years)	28 ± 1	42 ± 4	43 ± 7
Male (M)/Female (F)	14 M/43F	1 M/16F	2 M/7F
Body Mass Index (kg/m^2^)	27 ± 1	26 ± 1	28 ± 2
Ascending colon (cm)	21 ± 1	25 ± 2	23 ± 3
Transverse colon (cm)	38 ± 1	52 ± 3	39 ± 3
Descending colon (cm)	26 ± 1	33 ± 1	23 ± 2
Sigmoid‐rectum colon (cm)	43 ± 1	52 ± 3	44 ± 3
Total colon (cm)	127 ± 2	162 ± 6	129 ± 6

### 3.1 Colon Length in the Fasting Condition

**FIGURE 2:**
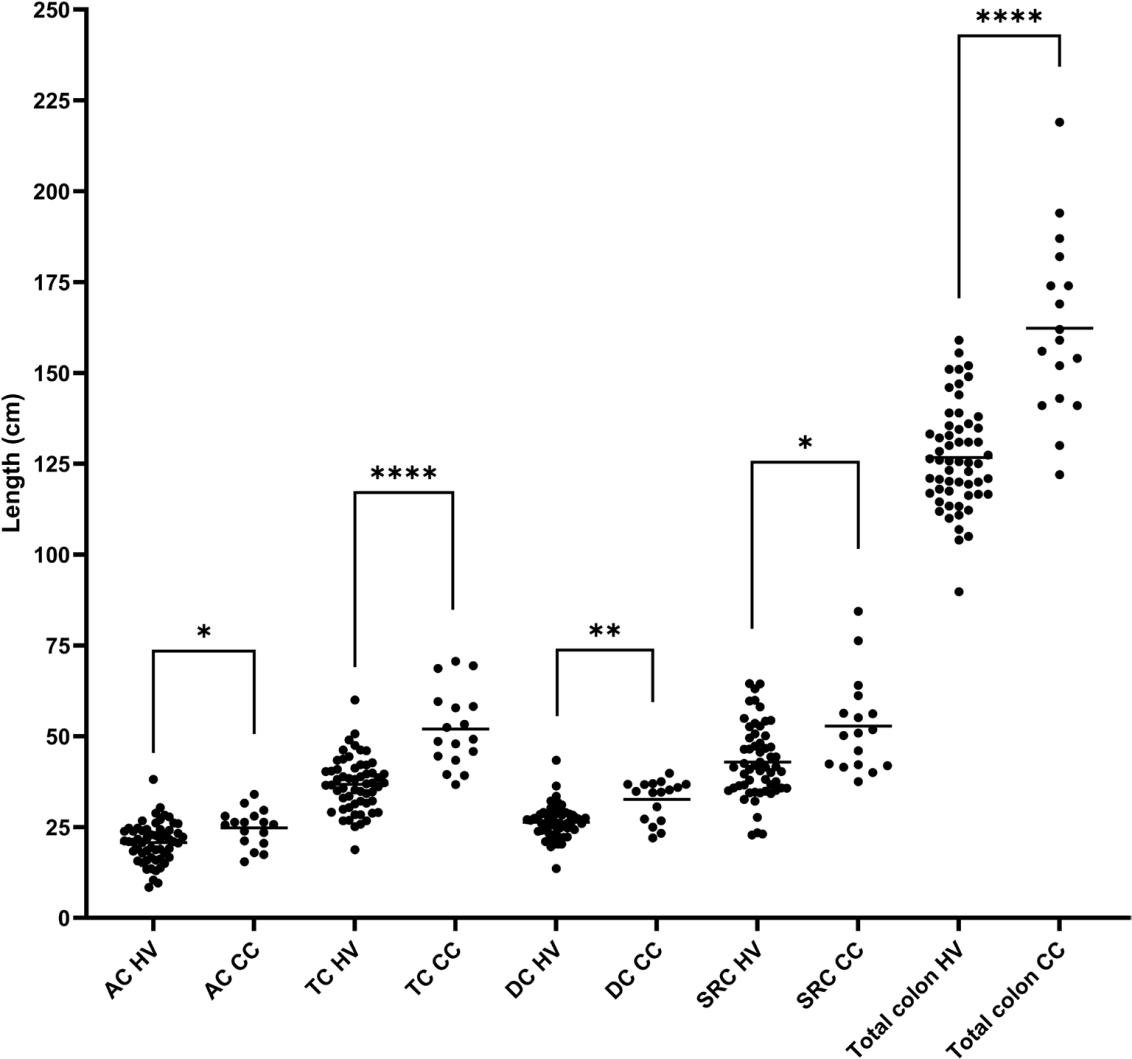
Colon length of adults with chronic constipation (CC, *n* = 17) and adult healthy volunteers (HV, *n* = 57). AC, ascending colon; TC, transverse colon; DC, descending colon; and SRC, sigmoid rectum colon. Statistical significance of differences between CC and HV is indicated in the figure by asterisks corresponding to *p* values: *p < 0.05, **p < 0.01, ****p < 0.0001.

The colon length normative values for HV are shown in (Table 1). Based on these measurements, the Upper Limit of Normal for total colon length is 154.5 cm. The comparison of colon length between patients with CC and HV demonstrated significant differences in both total and segmental measurements (Figure 2). The total colon length was significantly longer in the CC group 162 ± 6 cm, compared to HV 127 ± 2 cm (*p* < 0.0001), with 10 of 17 patients with CC having an abnormally long colon, that is, longer than the Upper Limit of Normal. Segmental analysis showed that the AC was significantly longer in CC 25 ± 1 cm compared with HV 21 ± 1 cm (*p* < 0.05). A greater difference was observed in the TC, where CC showed significantly increased length 52 ± 3 cm relative to HV 38 ± 1 cm (*p* < 0.001). Similarly, DC length was significantly longer in CC 33 ± 1 cm than in HV 26 ± 1 cm (*p* < 0.01). The SRC also demonstrated a statistically significant increase in length in CC 52 ± 3 cm compared with HV 43 ± 1 cm (*p* < 0.05). The spread of colon length values was greater for CC than for HV. The mean colon length values for each segment and total colon length are listed in (Table 1).

**FIGURE 3:**
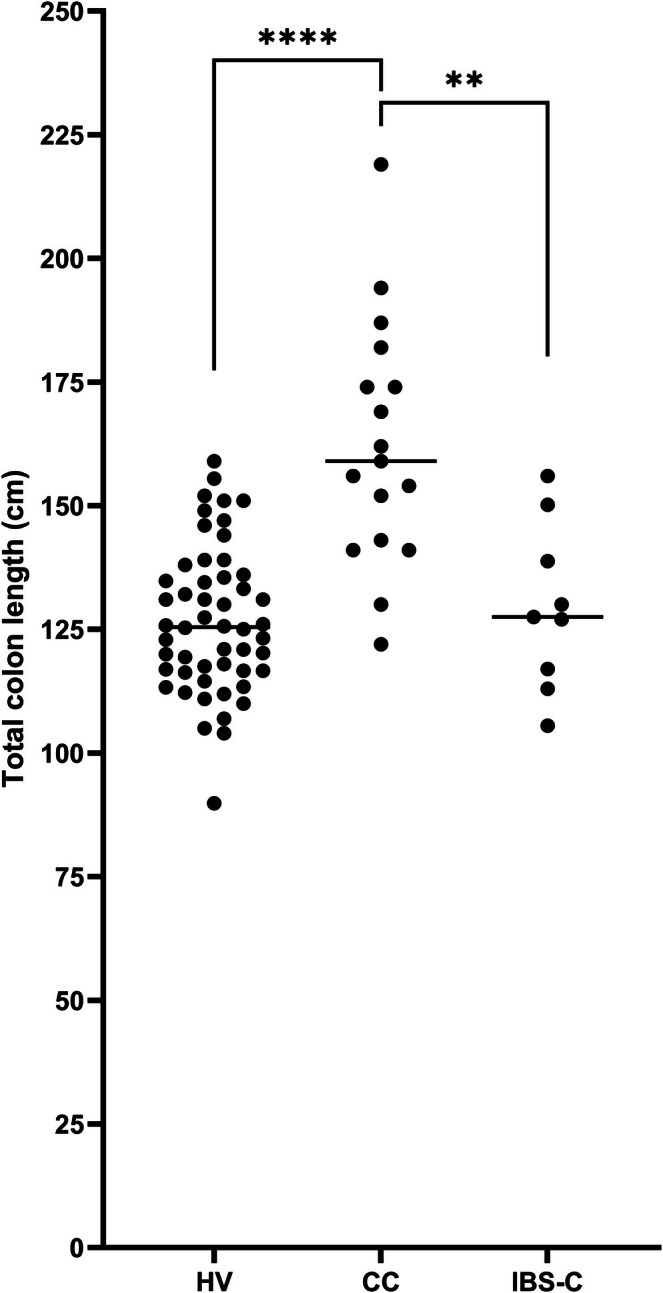
Total colon length for *n* = 57 healthy adult volunteers (HV), *n* = 17 adults with chronic constipation (CC), and *n* = 9 adults with irritable bowel syndrome with constipation (IBS‐C). Statistical significance is indicated in the figure by asterisks corresponding to *p* values: **p < 0.01, ****p < 0.0001.

Mean colon length for IBS‐C (129 ± 6) cm was not different from HVs (127 ± 2), *p* = 0.6347, but it was significantly shorter compared to CC 162 ± 6 cm (*p* = 0.0015) (Figure 3). The mean colon length values for each segment and total colon length for the people with IBS‐C are also listed in (Table 1).

**FIGURE 4:**
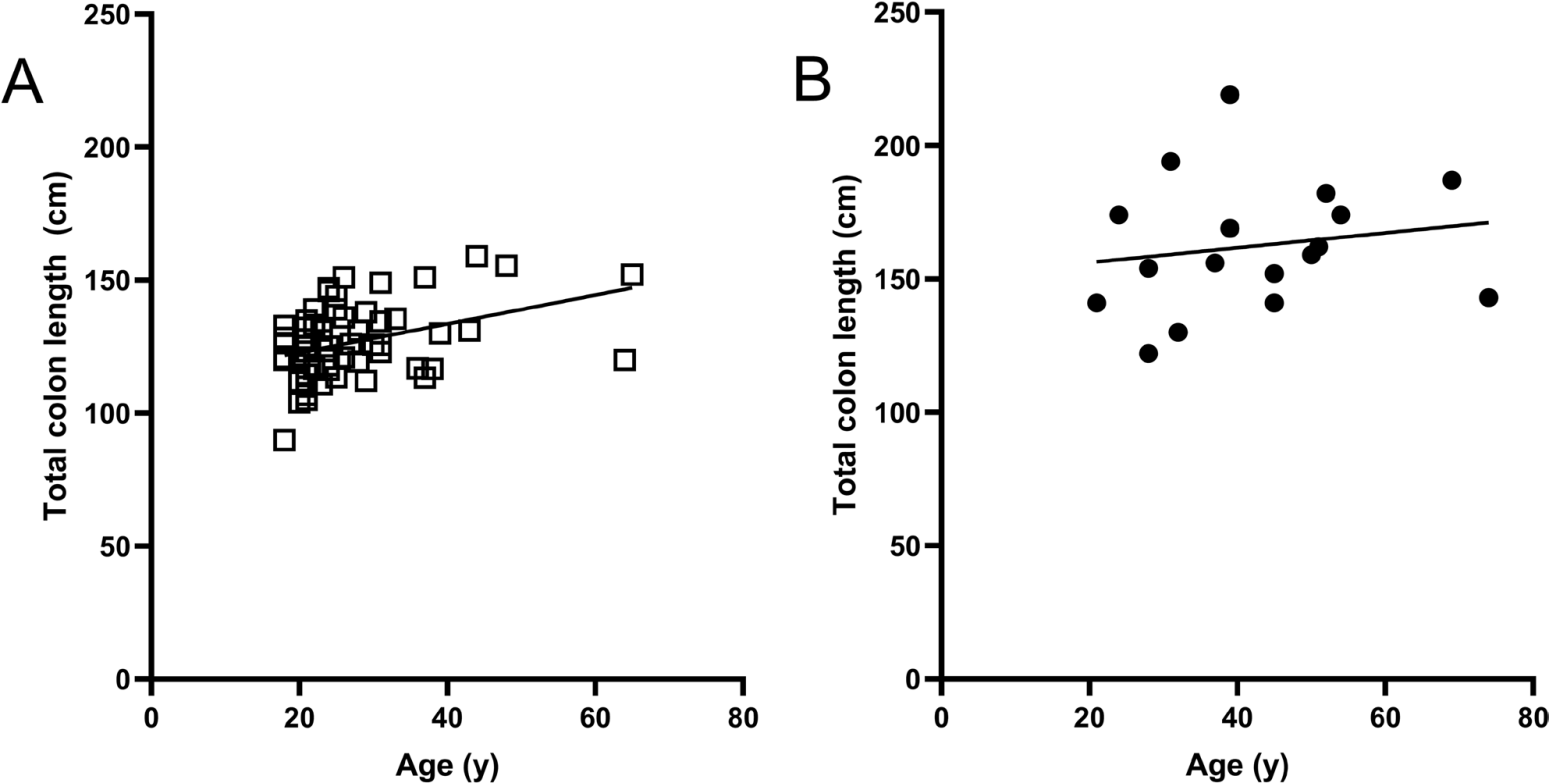
Relationship between total colon length and age for: (A) *n* = 57 healthy volunteers (linear regression *R* ^2^ = 0.15, *p* = 0.0033) and (B) *n* = 17 patients with chronic constipation (linear regression *R* ^2^ = 0.03, *p* < 0.52).

Total colon length showed a modest but significant positive correlation with age (Figure 4), for the 57 participants in the HV group (R^2^ = 0.15, *p* = 0.0033) but not for the patients. There were no significant correlations between total colon length and height, weight, and BMI for any of the three groups, nor for all the data pooled together.

A sub‐analysis by participants' sex for each group is shown in Table S1. The female colon was generally longer than the male colon, particularly the AC and TC segments, though small numbers did not allow meaningful statistical comparisons between male and female colon lengths. Pooling all the data from HV and patients for all three groups, for a total of 17 male and 66 female participants, shows that the TC was 19% longer in females compared to male participants, *p* = 0.0036.

### 3.2 Colon Length Response to an Osmotic Laxative Challenge

**FIGURE 5:**
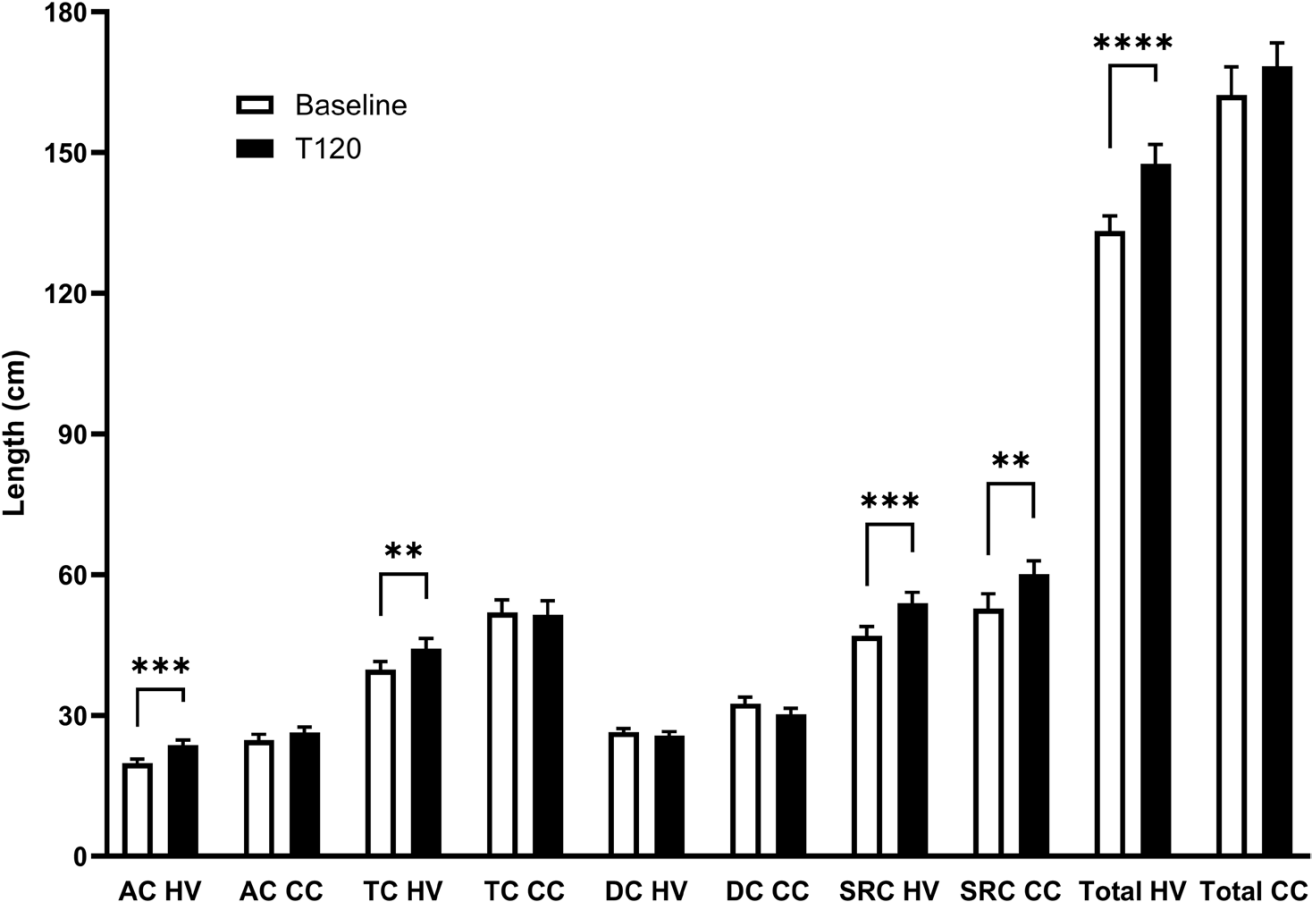
Colon length response to macrogol challenge at baseline and 120 min after ingestion in patients with chronic constipation (CC, *n* = 17) and healthy volunteers (HV, *n* = 22). AC, ascending colon; TC, transverse colon; DC, descending colon; SRC, sigmoid rectum colon. **p < 0.01, ***p < 0.001, ****p < 0.0001.

**FIGURE 6:**
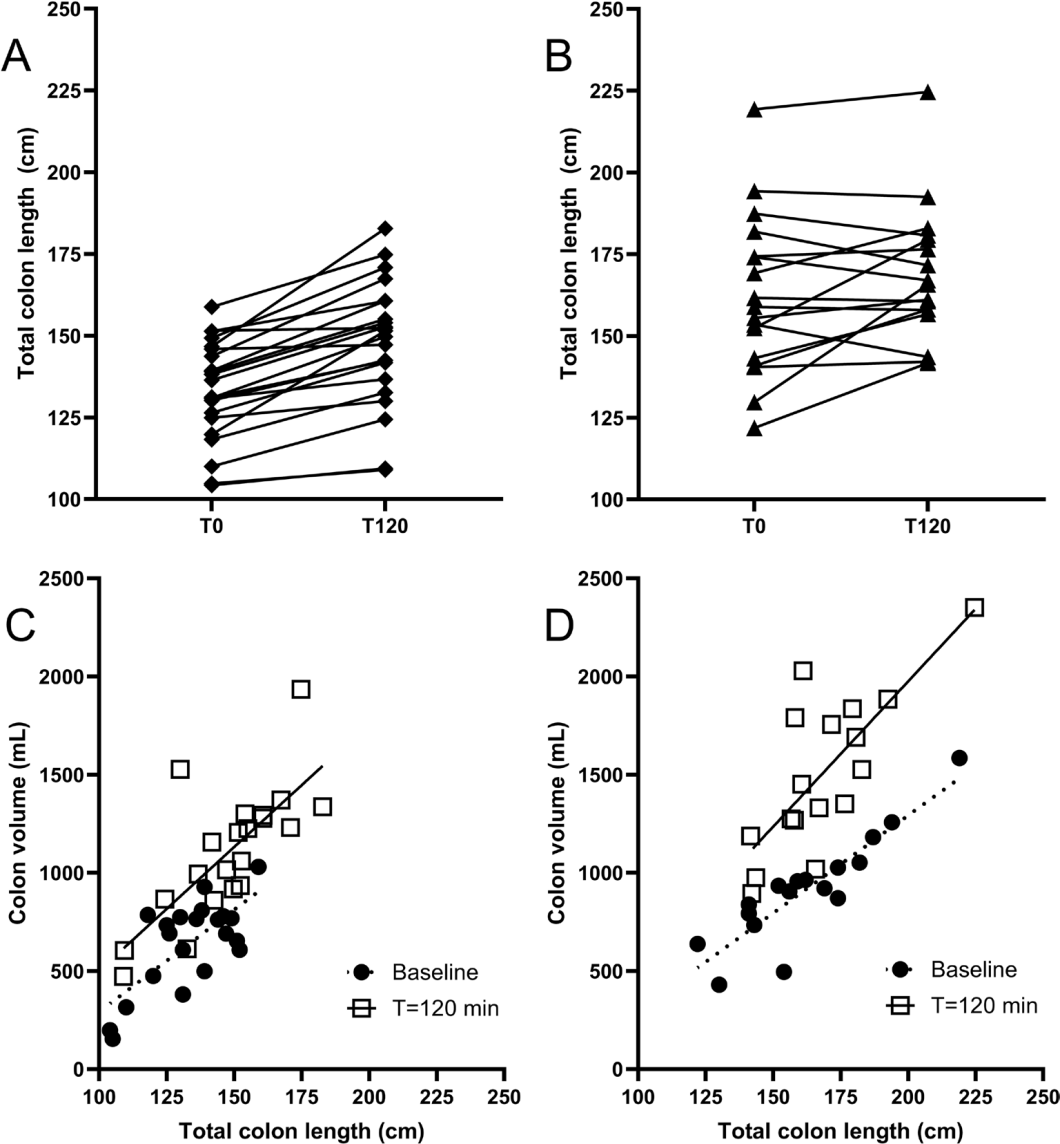
Individual values for Total colon length at baseline T0 and after osmotic laxative challenge T120 for (A) *n* = 22 adult healthy volunteers (HV) and (B) for *n* = 17 patients with chronic constipation (CC). The solid lines connect the values for each participant. Panels (C) for HV and (D) for CC show the individual total colon length plotted against the corresponding total colon volume. The solid dots represent the values at the fasted baseline, and the open squares represent the values 120 min after ingestion of an oral osmotic laxative challenge. The dotted and solid lines represent the respective linear regression fits.

Following the ingestion of macrogol, HV showed a significant total colon elongation from 133 ± 3 to 148 ± 4 cm in response to the macrogol challenge (*p* < 0.0001). In the HVs, the AC showed a significant increase from baseline 20 ± 1 to 24 ± 1 cm at 120 min (*p* = 0.0002), and so did the TC from 40 ± 2 cm to 44 ± 2 cm, respectively (*p* = 0.0035), and the SRC from 47 ± 2 to 54 ± 2 cm (*p* = 0.0004). No significant change was detected in the HV's DC. By contrast, patients with CC showed no significant changes in total colon length (from baseline 162 ± 6 to 168 ± 5 cm after intervention, *p* = 0.0768) or in colon segments in the time frame 0–2 h, except for a modest increase in length in the SRC from 53 ± 3 cm at baseline to 60 ± 3 cm, *p* = 0.0046 (Figure 5).

(Figure 6), shows the change in colon length for each participant at T120 after the osmotic laxative challenge (Figure 6A). Shows that almost all the HVs increase colon length at T120, but this is not the case for the patients with CC in (Figure 6B), particularly for those with the longer colon at baseline. Plots of each participant's colon volume against the corresponding colon length at baseline and after macrogol challenge are shown in (Figure 6). In HVs (Figure 6C), the osmotic laxative challenge induced a colon volume increase of 20% greater than colon length. By contrast, in patients with CC, the increase in colon volume induced by the osmotic laxative challenge was approximately 50% greater than the corresponding increase in colon length (Figure 6D). In more detail, the linear regression for HVs had a slope of 10.39 with R^2^ = 0.1 (*p* = 0.0003) at baseline and a slope of 12.60 with R^2^ = 0.54 (*p* = 0.0001) 120 min after macrogol intervention. In CC, the linear regression had a slope of 9.95 with R^2^ = 0.79 (*p* < 0.0001) at baseline and a higher slope of 14.80 with R^2^ = 0.58 (*p* = 0.004) 120 min after macrogol intervention.

### 3.3 Intraoperator and Within‐Subject Variability

The intraoperator variability was low for all colon segments and total colon length measurements, with CoVs varying between 2% and 8% (Table 2). The variability of fasting baseline colon length for each subject assessed across four separate, repeated visits was higher than intraoperator variability and ranged between 9% and 22%. The more distal segments of the colon showed higher variability across repeated visits.

**TABLE 2:** Assessment of intraoperator variability of colon length measurement performed on one participant measured on six different occasions, and of within‐subject variability performed on eight patients who had one MRI study repeated every week for 4 weeks. The data are presented as mean ± SD and as coefficient of variation (CoV).

	Intraoperator		Within‐subject	
	Length (cm)	CoV (%)	Length (cm)	CoV (%)
Ascending colon	26 ± 1	4	21 ± 2	10
Transverse colon	30 ± 1	2	29 ± 3	11
Descending colon	36 ± 3	8	26 ± 5	19
Sigmoid‐rectum colon	31 ± 1	4	39 ± 9	22
Total colon	123 ± 2	2	121 ± 11	9

## 4 Discussion

The length of the adult, undisturbed, unprepared colon in CC and its dynamic response to an osmotic laxative challenge have been measured for the first time from MRI scans and compared with HV and IBS‐C. This study also provides normative MRI‐based values of adult colon length from the largest dataset measured to date.

At baseline, patients with CC showed significantly longer mean total colons compared with HVs. Elongation was observed across all colonic segments, with the most pronounced mean differences in the TC and SRC. These findings support previous work that has associated colonic elongation with CC and may reflect chronic structural adaptation due to prolonged stool retention or neuromuscular dysregulation [24]. Looking at the individual values in (Figure 2), rather than considering the mean values, 10 out of 17 CC patients (59%) had total colon length larger than the HV mean plus 2 standard deviations. Further work will be needed to explore the relevance of the presence or absence of an abnormally elongated colon to symptom phenotype. Colon length and redundancies have been associated with bowel symptoms and slower gut transit [7, 10].

Colon length values measured in this study for HV, with a total colon length mean of 127 cm, are longer than the mean values reported in the skeletonization MRI (95 cm) and electromagnetic capsule tracking (99 cm) paper [16]. This could be due to the different measurement techniques used or the predominantly female group in our study compared to male‐only participants in the other study. A recently published meta‐analysis [11], calculated the mean adult total colon length to be 148 cm. This is longer than the values measured here but includes studies using colon inflation with contrast media and cadaveric studies, which are known to show longer colons than normal in situ undisturbed morphology [9, 12].

The finding that colon length is longer in female than in male participants is in keeping with other reports for total colon length [25], particularly for having a longer transverse colon [10], though the small numbers of the sub‐analyses here do not allow strong inferences.

Following the challenge of the macrogol intervention, the HV colon showed the ability to acutely accommodate the influx of fluid by significantly increasing colon length as well as colon volume, with the latter demonstrated previously in the RECLAIM study [18]. By contrast, the macrogol challenge did not induce significant total colon length changes in the CC group, particularly for the CC colons that were the longest at baseline, while it is known from the previous study that colon volumes increase after the challenge [18]. These data show a blunted longitudinal accommodation response to luminal distension, which may reflect a colon already elongated by the condition and not capable of additional longitudinal stretch. This is further evidenced by plotting colon volumes against colon length before and after the challenge. This analysis showed that in the CC group, postintervention colon volume increased at a rate approximately 50% higher than for length, suggesting a predominant radial (i.e., axial) expansion. In HVs, the rate of increase in volume with length was lower, at approximately 20%, indicating a more balanced ability to stretch in both dimensions to accommodate the extra fluid. This divergence in distension mechanics may reflect altered tissue properties or reduced muscular tone in the CC group and highlights the potential value of combining length and volume metrics when assessing colonic distensibility.

Albeit with small numbers *n* = 9, the total colon length comparison against IBS‐C patients was particularly interesting, as it showed that mean colon length for people with IBS‐C was the same as that of HVs, in keeping with the accepted construct that the main difference between HV and IBS‐C is hypersensitivity with or without dysmotility leading to pain [26], rather than differences in anatomy.

The difference in colon length between constipation types raises questions about the role of colonic wall mechanics in CC and suggests that longitudinal stretch resistance may be a factor in disease pathophysiology. Future studies may benefit from exploring associations between segmental compliance and clinical outcomes, as well as assessing whether these structural traits could potentially serve as imaging biomarkers for constipation subtyping or treatment response. If confirmed, pathophysiological distinctions in colon morphology may increase the possible value of imaging‐based assessments in differentiating between subtypes of constipation.

Colon length intraoperator reproducibility was good. Variability of colon length on repeated studies, however, was higher, particularly for the more distal regions (DC and SRC). These regions are likely more variable because of increased tortuosity and also because they may be influenced by the timing of defecation in relation to the scans, a feature that is hard to control.

With regards to the measurement technique, the direct 3D tracing on the image stacks appeared to be quicker and less laborious than 3D skeletonization methods [17], where the algorithms can also branch out incorrectly and affect segmental length depending on the quality of the images and continuity of the segmentations. The high intraoperator and intrasubject variability of the colon length measurements was good, and the actual measurement on the image analysis platform utilized is not difficult, allowing for the tracing of the centerline for the colon segments with tailored navigation of individual anatomical variation. The coefficient of variation was reasonably low. These observations support the feasibility of applying this 3D MRI‐based method in future studies and clinical assessments.

Limitations of this study were the retrospective nature of the data and the fact that the operator was not blinded. As another limitation, image quality occasionally varied due to patient‐specific factors such as bowel gas or anatomy, but the visualization of the bowel in 3D facilitated the operator's navigation through the organ and the measurement. The retrospective data were also collected on a range of different MRI scanners with different scan protocols, which could also have influenced the measurements.

This study highlights the value of using MRI for assessing colon length and dynamic responses in CC. Future research should focus on larger and more diverse cohorts to confirm these findings and explore demographic influences and relationships with symptoms. Incorporating measurements of both colon volume and length over time may offer a more complete view of colonic compliance. Development of semi‐automated segmentation tools could improve accuracy and reduce operator bias. Longitudinal studies and integration with motility metrics may further clarify the clinical relevance of these structural changes and support MRI‐based biomarkers in guiding treatment strategies and evaluating response to drugs designed to alter colonic motility.

## 5 Conclusion

This study shows that MRI imaging can be used to reliably measure the length of the colon in a physiological, unprepared state and demonstrate colon length changes with disease and intervention. The ability to carry out these measurements will improve our understanding of gut disease physiopathology and, following further work, it could also prove useful for differentiating phenotypes, monitoring the mode of action of pharmaceutical agents.

## Author Contributions

F.A. and LM: study concept and design, acquisition and analysis of data; statistical analysis, drafting the manuscript. L.M., M.A.T., and G.P.A.: PhD project supervision. S.K., C.L.H, V.W.‐S, D.A., S.T., and P.A.G.: acquisition and analysis of data. R.C.S., M.C., S.M.S., and V‐W‐S.: patient selection and inclusion. I.N. and A.M.: image analysis platform. F.A., S.M., C.L.H., V.W.‐S., S.T., D.A., I.N., A.M., S.M.S., M.B., P.A.G., M.A.T, G.P.A., M.C, R.C.S., and L.M. interpretation of data; critical revision of the manuscript for important intellectual content. All authors have read and agreed to the published version of the manuscript.

## Funding

This work was supported by King Saud University, the National Institute for Health and Care Research, and the Medical Research Council.

## Ethics Statement

The data were retrieved retrospectively from studies that were approved by the NHS and institutional review boards: RECLAIM study approval number 17/EM/0032; MAGIC study approval number 17/WM/0049; APPLE study approval number A13102015; KIWI study approval number A200317; MRLENGH study approval number FMHS 204–0223; EFIGI study, approval number 17/EM/0277. All participants had provided written informed consent. The current study was conducted in accordance with the ethical principles expressed by the Research Ethics Committees (NHS REC and University of Nottingham REC).

## Conflicts of Interest

A.M. is the founder and CEO of Motilent. I.N. is employed by Motilent.

## Supporting information


**Table S1:** Participants' demographics and colon length measurements were divided by sex. Values are presented as mean ± SEM. Statistical comparisons between males and females were not made when the number of male participants was too small. *p* values in this table are not corrected for multiple comparisons.

## Data Availability

The data that support the findings of this study are available from the corresponding author upon reasonable request.
