# Supplementary material for: Length of the Adult Human Colon in Health and Constipation Measured Using Magnetic Resonance Imaging
Source: Neurogastroenterol Motil. 2025 Dec 2;38(6):e70215. doi: 10.1111/nmo.70215 (PMC13244114; doi:10.1111/nmo.70215)
Supplement: Supplementary file 1 — Table S1: Participants' demographics and colon length measurements were divided by sex. Values are presented as mean ± SEM. Statistical comparisons between males and females were not made when the number of male participants was too small. p values in this table are not corrected for multiple comparisons. [file NMO-38-e70215-s001.docx]

**Supplementary Table S1:** Participants’ demographics and colon length measurements divided by sex. Values are presented as mean±SEM. Statistical comparisons between males and females were not made when the number of male participants was too small. P values in this table are not corrected for multiple comparisons.

| **Healthy volunteers** | **Male** | **Female** | **p** |
| --- | --- | --- | --- |
| n | 14 | 43 |  |
| Age (years) | 28±2 | 27±2 | 0.8722 |
| Body Mass Index (kg/m2) | 27±1 | 25±1 | 0.1103 |
| Ascending colon (cm) | 19±2 | 21±1 | 0.4430 |
| Transverse colon (cm) | 34±1 | 38±1 | **0.0490** |
| Descending colon (cm) | 28±1 | 26±1 | 0.1982 |
| Sigmoid-rectum colon (cm) | 42±3 | 43±1 | 0.7321 |
| Total colon (cm) | 123±3 | 128±2 | 0.5915 |
| **Chronic constipation** | **Male** | **Female** | **p** |
| n | 1 | 16 |  |
| Age (years) | 45 | 42±4 | - |
| Body Mass Index (kg/m2) | 29 | 26±1 | - |
| Ascending colon (cm) | 21 | 25±1 | - |
| Transverse colon (cm) | 46 | 52±3 | - |
| Descending colon (cm) | 35 | 32±1 | - |
| Sigmoid-rectum colon (cm) | 51 | 53±3 | - |
| Total colon (cm) | 152 | 163±6 | - |
| **Irritable bowel syndrome with constipation** | **Male** | **Female** | **p** |
| n | 2 | 7 |  |
| Age (years) | 29±10 | 47±8 | - |
| Body Mass Index (kg/m2) | 25±7 | 30±1 | - |
| Ascending colon (cm) | 20±1 | 24±4 | - |
| Transverse colon (cm) | 34±7 | 40±4 | - |
| Descending colon (cm) | 27±7 | 22±2 | - |
| Sigmoid-rectum colon (cm) | 45±2 | 44±3 | - |
| Total colon (cm) | 126±13 | 130±7 | - |
